# Changes in Size and Age of Chinook Salmon *Oncorhynchus tshawytscha* Returning to Alaska

**DOI:** 10.1371/journal.pone.0130184

**Published:** 2015-06-19

**Authors:** Bert Lewis, W. Stewart Grant, Richard E. Brenner, Toshihide Hamazaki

**Affiliations:** 1 Alaska Department of Fish and Game, Commercial Fisheries Division, Anchorage, Alaska, United States of America; 2 Alaska Department of Fish and Game, Commercial Fisheries Division, Juneau, Alaska, United States of America

## Abstract

The average sizes of Pacific salmon have declined in some areas in the Northeast Pacific over the past few decades, but the extent and geographic distribution of these declines in Alaska is uncertain. Here, we used regression analyses to quantify decadal trends in length and age at maturity in ten datasets from commercial harvests, weirs, and spawner abundance surveys of Chinook salmon *Oncorhynchus tshawytscha* throughout Alaska. We found that on average these fish have become smaller over the past 30 years (~6 generations), because of a decline in the predominant age at maturity and because of a decrease in age-specific length. The proportion of older and larger 4-ocean age fish in the population declined significantly (*P* < 0.05) in all stocks examined by return year or brood year. Our analyses also indicated that the age-specific lengths of 4-ocean fish (9 of 10 stocks) and of 3-ocean fish (5 of 10 stocks) have declined significantly (*P* < 0.05). Size-selective harvest may be driving earlier maturation and declines in size, but the evidence is not conclusive, and additional factors, such as ocean conditions or competitive interactions with other species of salmon, may also be responsible. Regardless of the cause, these wide-spread phenotypic shifts influence fecundity and population abundance, and ultimately may put populations and associated fisheries at risk of decline.

## Introduction

Size and age at maturity are important life-history traits for Pacific salmon (*Oncorhynchus* spp.), reflecting an assortment of evolutionary and ecological influences [1]. The average sizes of Pacific salmon have declined in some areas in the Northeast Pacific but the geographic distribution and species-specific extent of these declines in Alaska is unknown. Several studies have shown that individuals in many areas are returning to spawn at an earlier age and at smaller sizes over the last few decades [2–5]. A reduction in size at maturity is important because it may influence individual reproductive potential and population fitness. Larger females, for example, have higher fecundities allowing them to spawn with more than one male and to spread genetic risks [6]. Larger females also have larger eggs which increases survival [7]. Size and fecundity of salmon is determined by duration in the ocean where most growth occurs. The number of years these anadromous fish spend in the ocean is determined by tradeoffs between foraging time to grow large enough for gonad development and energetically costly spawning migrations, and the increased risk of mortality the longer a fish remains in the ocean [6,8]. Hence, size and age at maturity are likely influenced by variables affecting growth and survival, including competition, food availability, predation, disease, temperature, and harvest intensity [9–13]. The relative importance of these factors in shaping life-history variability is largely unknown.

**Fig 1:**
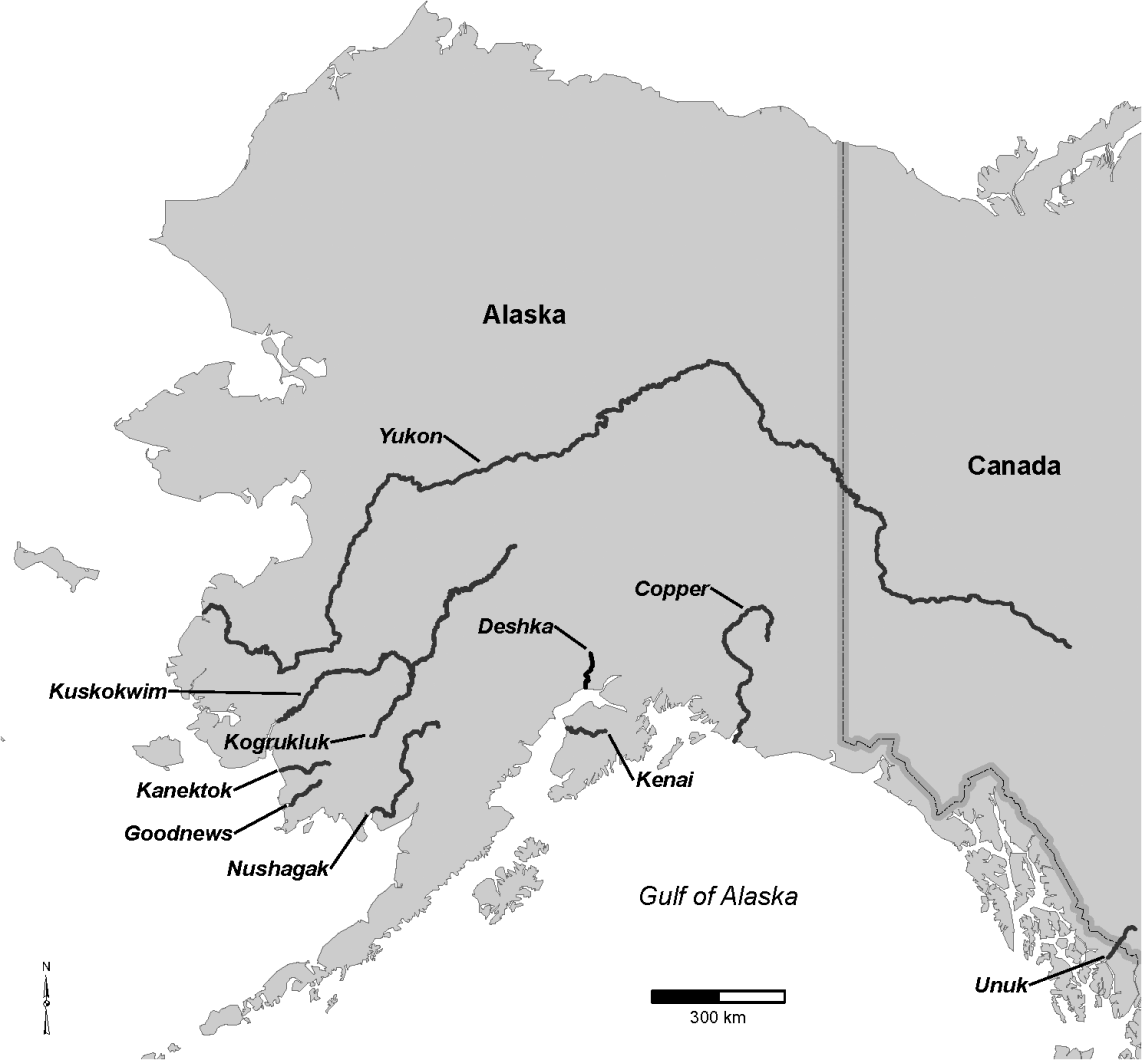
Map of Alaska with locations of Chinook salmon populations discussed in this study.

The goal of this study was to assess trends in length and age of maturation in ten widely scattered stocks of Chinook salmon *O*. *tshawytscha* in Alaska (Fig 1). While shifts in size and age at maturity have been noted in a few Alaska populations [14–16], it is unknown if these changes occur in other Chinook populations on a broader scale. Chinook salmon have variable life histories, spending 0–2 years in fresh water and 1–4 years (or more) in the ocean migrating thousands of kilometers in the North Pacific and (or) Bering Sea before returning to natal rivers to spawn [1]. Size and age at maturity of Chinook salmon have been routinely collected for several decades as part of harvest and escapement (spawner abundance) monitoring throughout much of the geographic range of these fish. Harvest managers use these data to measure the progress and size of annual runs and to build models to forecast future returns. These datasets also provide a means of detecting size and age at maturity changes on decadal time scales and on large geographic scales.

Declines in Chinook abundances from recent historical highs in Alaska have been pervasive in the Northeast Pacific, particularly over the past decade [15,17,18]. The present study examines the dynamics of two life-history traits in these declining stocks to better understand the nature of the declines. We resolve trends in overall size into two components, size at age and age at maturity, and find that both variables have declined in the past few decades. We then document whether any of these trends reflect the locations of the spawning stocks or responses to the magnitude or methods of harvest in commercial fisheries. The examination of long-term trends in the ages and sizes of returning Alaska Chinook salmon is the first step to better understand the extent that ecological, environmental and (or) anthropogenic drivers might be influencing life-history traits in this species [19–22].

## Materials and Methods

### Age-length (AL) datasets

Alaska Department of Fish & Game (ADF&G) monitors the number, size, and ages of returning fish for many Alaska Chinook salmon stocks. We selected datasets for 10 stocks based on the duration and consistency of the time series, sample sizes, and data collection methods (Table 1). Most age and length (AL) data were collected from commercial gillnet harvests in marine waters near river mouths. These samples often include a mix of fish from different populations within the river drainage and, in some cases, outside the drainage (e.g. Kenai River). Samples from spawner abundance monitoring projects (weirs and carcass surveys) potentially had fewer, or different, size-selective biases than samples from commercial gillnet harvests. For example, samples collected at weirs were believed to include a broader range of size classes than those sampled from harvests by gillnets, which might not ensnare the smallest or largest sizes of Chinook salmon. Sampling methods did not change consistently over time; however, some changes in the fisheries did occur, such as changes in gillnet mesh sizes.

**Table 1:** Chinook salmon data sources, mean annual sample size per year, and relative exploitation rates by stock.

River system	Source of data	Gear (stretched mesh size inches)	N*yr^-1^	Exploitation
Yukon	Commercial Harvest	Gillnet (5.5–8.5)	649	High
Kuskokwim	Commercial Harvest	Gillnet (3–8.5)	146	Medium
Kogrukluk	Escapement	Weir	168	Medium
Kanektok	Commercial Harvest	Gillnet (<6)	187	High
Goodnews	Commercial Harvest	Gillnet (<6)	108	Low
Nushagak	Commercial Harvest	Gillnet (<5.5–unrestricted)	347	Low
Deshka	Escapement	Weir	145	Low
Kenai	Commercial Harvest	Gillnet (<6)	347	Medium
Copper	Commercial Harvest	Gillnet (<6–unrestricted)	421	High
Unuk	Escapement	Snag, dip-net, seine, carcass	225	Low

The AL datasets used in this study were collected using methods following Tobias et al. [23]. As part of basic fisheries monitoring, ADF&G personnel routinely measured mid-eye-to-fork length to the nearest millimeter with a measuring tape, or with a manual, or electronic, measuring board, depending on project and year (ADF&G staff personal communications). Fish age was estimated from scales with collection and aging protocols that were the same among years and locations. The age of a fish was denoted by the number of years spent in marine waters (ocean age). For example, a fish spending four years at sea and having four winter annuli in the ocean zone of the scale was designated as a 4-ocean fish. A small number of ocean-type Chinook salmon, fish which emigrate to sea as sub-yearlings, were excluded from the analyses. Data from 2-, 3- and 4-ocean fish, which represent the most of the population, were used in this study. We elected to use ocean age because Alaska Chinook salmon generally have a stream-type life history, with most spending a single year in freshwater before migrating into salt water.

Age-frequency distributions were estimated for all 10 datasets by return year (year a mature fish returned to spawn) and by brood year (year a fish was born). Brood-year returns were estimated by assigning numbers of fish by age-class of the total return back to their brood year. Estimates of total return (sum of harvests and spawner abundance) and brood-year calculations were available for only 5 of the 10 datasets. Age-class proportions by return year are a mix of fish from different brood years and, hence, may be biased by cohort abundance. Age-class frequencies by brood year provide a more accurate estimate of population age structure than age-class frequencies by return year.

We estimated exploitation rates to show the relative impact of fisheries on the mortality of adults from individual stocks. Exploitation rate for each stock was estimated as the sum of annual commercial, sport, subsistence, and personal-use harvests divided by the sum of all harvest and spawner abundance estimates for a given brood-year and then averaged across all years for which data were available. However, it was not possible to make accurate point estimates of exploitation rates in most cases, because harvest and spawner abundance reporting were inconsistent and in some cases uncertain. Therefore, stock-specific exploitation rates were qualitatively categorized as low (<40%), medium (40–50%), or high (>50%).

Fish were sampled from commercial harvests, weirs, spawning area carcass surveys, or spawner abundance surveys that used rod and reel, dip nets, or gillnets (Table 1). Five annual samples with sizes less than *n* = 15 for an age class at a location were excluded from the analysis. For fish sampled in gillnet fisheries, mesh size varied within and among years for most locations, but generally not at specific times that could be used to test for corresponding changes in the size of fish harvested (Table 1). Only the Kenai, Kanektok, and Goodnews fisheries consistently used a single mesh size less than 6 inches for the entire time series. The Kenai dataset was from commercial mixed-stock gillnet harvests of predominantly Kenai River Chinook salmon (three-year average stock composition of 69% Kenai River fish), but also included small portions of fish from the Kasilof River and other Cook Inlet stocks [24]. We also included datasets for two weirs (Kogrukluk and Deshka rivers) and the Unuk River spawner abundance monitoring project (consisting of carcass surveys, beach seining, snagging, dipnet).

Length and age analyses were not separated by sex, because sex was frequently estimated with unreliable external characteristics [25] in ocean-phase fish. A recent study of size and age at maturity for Chinook salmon in the Nushagak River found similar trends for females and males [16], and we assumed that our results reflected similar trends in males and females.

### Statistical analysis

We used linear regressions (α = 0.05) to identify temporal trends in length-at-age (average annual values) [26]. Age proportions by return year follow a multinomial distribution whereas proportions by brood year follow a Dirichlet distribution [27]. Hence, to identify trends in age-at-maturity we used logistic regression for age proportions by return year and beta regression for age proportions by brood year (α = 0.05) [28,29]. In all regression analyses, Year of return was the independent variable and length or age proportion was the dependent variable. After regressing the dependent variables against time we tested each dataset (α = 0.05) by examining residuals for normality (Shapiro-Wilk test), constant variance (graphical examination), and independence (Durbin-Watson test) [26]. Datasets showing auto-correlation (Durbin-Watson test) were transformed using a Cochrane-Orcutt transformation [30] before the regression analysis. We tested the hypothesis that the regression slopes of the length and age (by return and brood year) were significantly larger or smaller than 0. Statistical analyses were conducted in the R statistical package [31].

## Results

**Fig 2:**
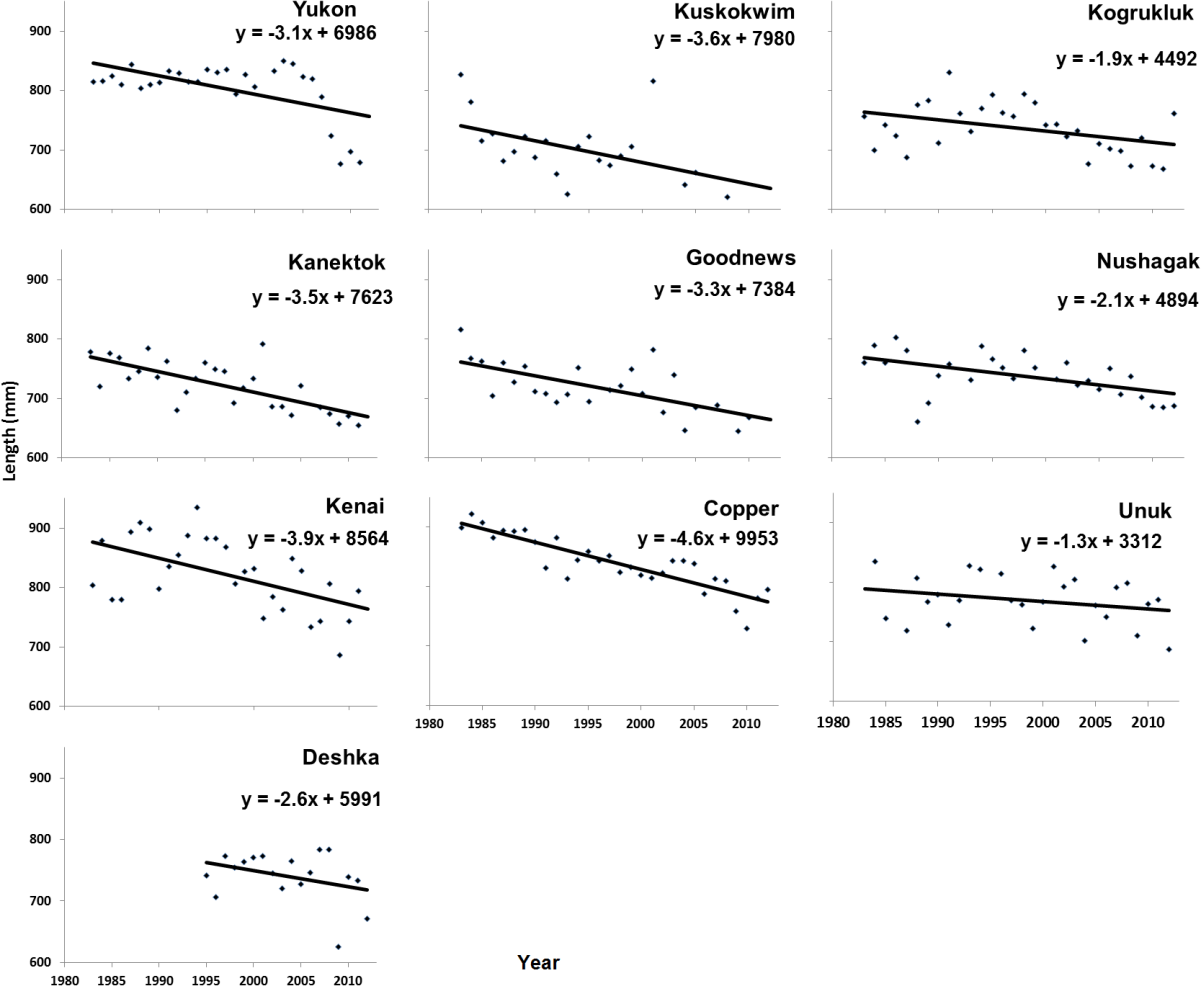
Linear regression of Chinook salmon mean annual length (mm) by stock and year.

The mean length of returning Chinook salmon varied considerably among and within areas over time (Fig 2). All of the regressions of the 10 river systems showed overall declines in size since 1983; however, the pattern of decline differed among areas (Fig 2). For example, the mean lengths of fish returning to the Yukon and Kogrukluk rivers were relatively stable until the early to mid-2000s, then declined rapidly. In the other areas, average length declined gradually since the early 1980s.

**Fig 3:**
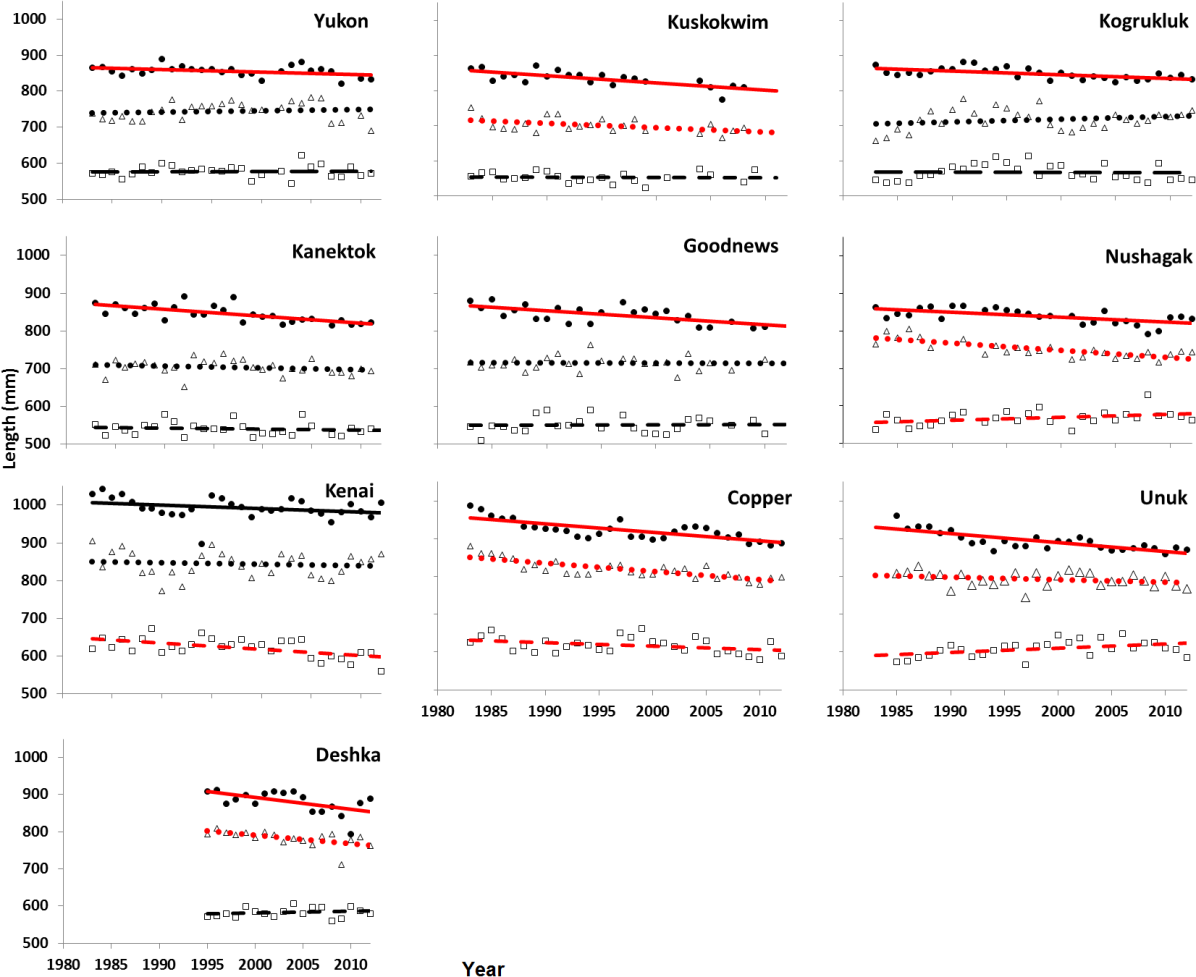
Linear regression of mean annual length (mm) Chinook salmon by stock, age class, and year. Closed circles and solid line = 4-ocean; triangles and dotted line = 3-ocean, open square and dashed line = 2-ocean. Red lines indicate slopes significantly different from zero (*P* <0.05).

We examined these overall trends in more detail by partitioning the length-at-age data by age class (2-, 3- and 4-ocean fish). Shapiro-Wilk tests indicated that 5 of the 30 mean length-at-age datasets departed significantly from a normal distribution. Graphical examination of residuals versus length did not provide evidence to reject the assumption of constant variances. About half of the length-at-age datasets showed significant temporal autocorrelation and these datasets were transformed to account for the autocorrelation. All of the regression slopes (expressed in mm*year^-1^) for average lengths of 4-ocean fish over time were negative (Fig 3), and 9 of 10 of these regression slopes were significantly less than 0 (*P* < 0.04) (with or without the Cochrane-Orcutt transformation for autocorrelation). For 3-ocean fish, slopes were negative in 8 of the 10 datasets, but only half of the stocks showed a significant trend in mean length (with or without the Cochrane-Orcutt transformation) since the early 1980s. No overall trend in length was found for 2-ocean fish, with 4 stocks deviating significantly from zero: 2 positive and 2 negative (*P* < 0.05) (Table 2). For this age component, none of the 5 most northern stocks had slopes that deviated significantly from zero.

**Table 2:** Linear regression of mean annual length (mm) of mature Chinook salmon in Alaska by return year from 1983 to 2012.

River system Age 4-Ocean	Slope	Upper 95% CI	*F*	Errord.f.	*P*
Yukon	-0.71	0.69	4.47	25	**0.04**
Kuskokwim	-2.08	0.88	14.98	20	**<0.01**
Kogrukluk	-1.08	0.53	17.48	28	**<0.01**
Kanektok	-1.84	0.76	24.78	26	**<0.01**
Goodnews	-1.83	0.93	16.74	23	**<0.00**
Nushagak	-1.38	0.68	17.62	26	**<0.01**
Deshka	-3.22	2.51	7.41	16	**0.02**
Kenai	-0.98	1.16	3.02	28	0.09
Copper	-2.28	0.75	39.08	28	**<0.01**
Unuk	-2.80	1.07	29.05	26	**<0.01**
3-Ocean					
Yukon	0.31	1.20	0.28	25	0.60
Kuskokwim	-1.27	1.08	24.19	20	**0.02**
Kogrukluk	0.76	1.27	1.50	28	0.23
Kanektok	-0.45	0.91	1.04	26	0.32
Goodnews	-0.03	1.08	0.00	22	0.96
Nushagak	-1.93	0.67	35.67	24	**<0.01**
Deshka	-2.28	1.77	7.43	16	**0.02**
Kenai	-0.38	1.44	0.30	28	0.59
Copper	-2.23	0.63	52.13	28	**<0.01**
Unuk	-1.15	0.72	10.83	26	**<0.01**
2-Ocean					
Yukon	-0.02	0.79	0.00	25	0.97
Kuskokwim	-0.07	0.91	0.03	19	0.87
Kogrukluk	-0.13	1.00	0.07	28	0.80
Kanektok	-0.31	0.78	0.65	26	0.43
Goodnews	0.11	1.11	0.04	23	0.85
Nushagak	0.84	0.79	4.70	26	**0.04**
Deshka	0.45	1.27	14.21	28	0.46
Kenai	-1.68	0.82	0.57	16	**0.00**
Copper	-0.95	0.85	5.10	28	**0.03**
Unuk	0.95	0.89	4.76	27	**0.04**

Bold indicates slope (mm/year) is significantly (*P* < 0.05) different from 0.

**Fig 4:**
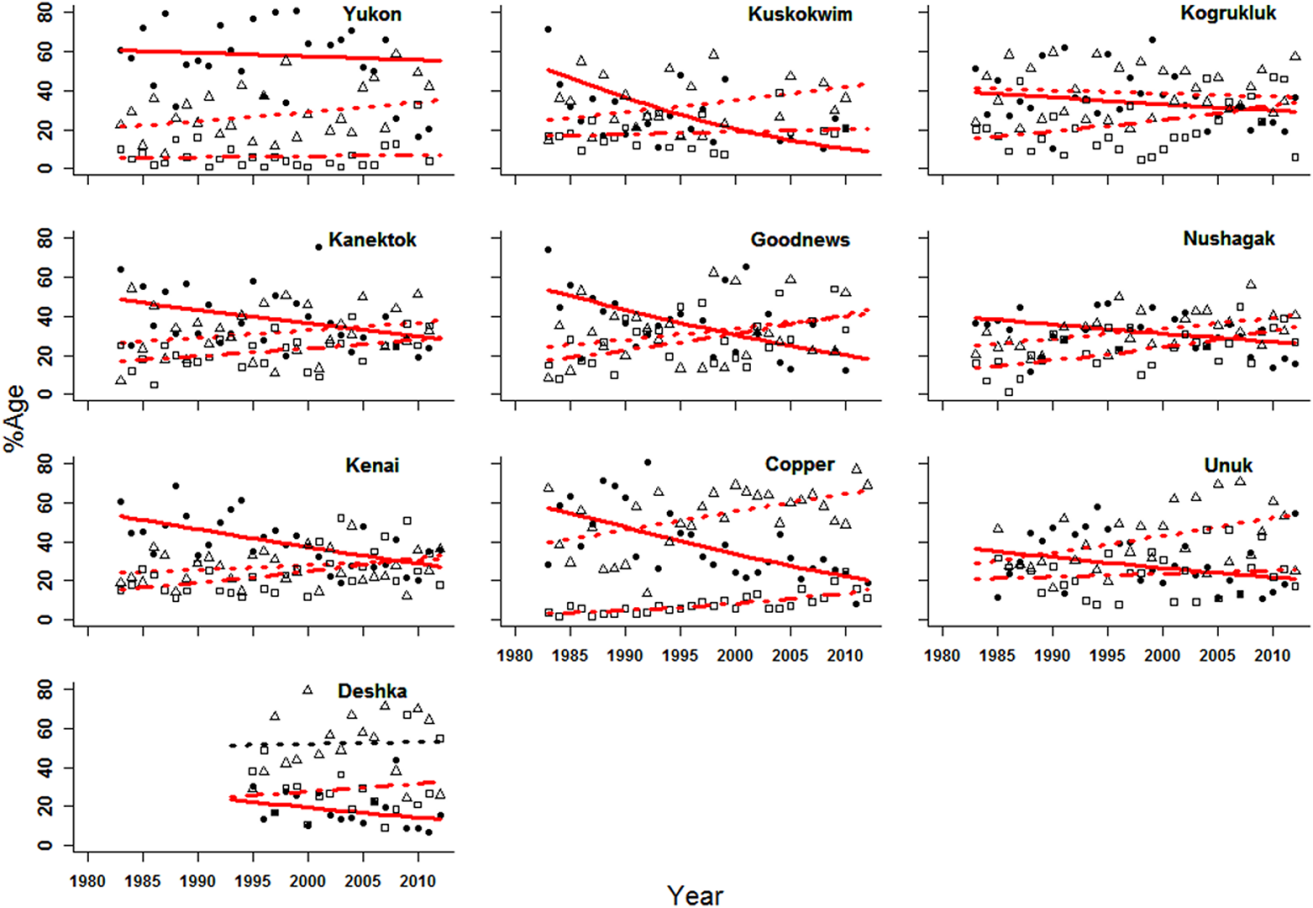
Logistic regression of proportion by return year of Chinook salmon by stock, age class, and year. Closed circles and solid line = 4-ocean; open triangle and dotted line = 3-ocean, open square and dashed line = 2-ocean. Red lines indicate slopes are significantly different from zero (*P* <0.01).

**Fig 5:**
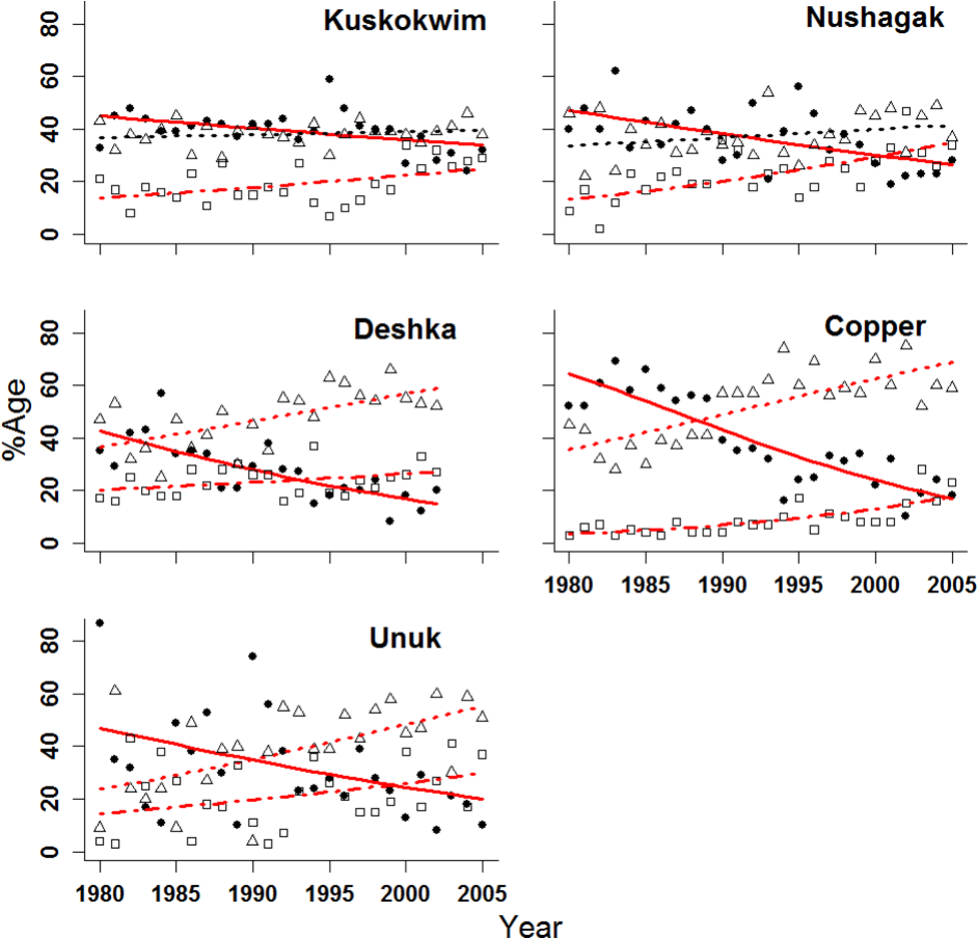
Beta regression of proportion by brood year of Chinook salmon stock, stock, age class and year. Closed circles and solid line = 4-ocean; open triangle and dotted line = 3-ocean, open square and dashed line = 2-ocean. Red lines indicate slopes significantly different from zero (*P* <0.05).

In addition to analyzing length-at-age of returning Chinook salmon, we examined temporal patterns in the relative frequencies of 2-, 3- and 4-ocean fish. The proportion of 4-ocean fish, by both return and brood year, declined significantly in all cases over the 30-year time series (Figs 4 and 5). All of the slopes (expressed in log(odds)year^-1^) of the proportions of 4-ocean fish by return year were negative and significant (*P* < 0.02) (Table 3). In many cases, the proportion of 3-ocean fish exceeded 4-ocean fish by return year at the end of the time series (Kuskokwim, Kanektok, Goodnews, Kenai, Nushagak, Copper, Unuk, and Deshka rivers). The slopes of 9 of 10 of the proportion of 3-ocean Chinook salmon by return year were positive, and 9 significantly (*P* < 0.01) increased through time (Table 3, Fig 4). Additionally, the slopes of all of the proportions of 2-ocean Chinook salmon by return year were positive and significantly increased (*P* < 0.01) through time (Table 3, Fig 4).

**Table 3:** Logistic regression of the proportions of 4-, 3- and 2-ocean aged Chinook salmon returning to spawning areas from 1983 to 2012.

River system Age 4-ocean	Slope	SE	*Z*	*P*
Yukon	-0.008	0.001	-5.95	**<0.01**
Kuskokwim	-0.081	0.004	-27.62	**<0.01**
Kogrukluk	-0.015	0.002	-7.46	**<0.01**
Kanektok	-0.029	0.002	-14.94	**<0.01**
Good News	-0.021	0.001	-14.73	**<0.01**
Nushagak	-0.038	0.001	-25.34	**<0.01**
Deshka	-0.036	0.006	-5.71	**<0.01**
Kenai	-0.056	0.003	-20.55	**<0.01**
Copper	-0.057	0.001	-54.44	**<0.01**
Unuk	-0.027	0.002	-12.20	**<0.01**
3-ocean				
Yukon	0.023	0.001	-54.44	**<0.01**
Kuskokwim	0.029	0.002	11.93	**<0.01**
Kogrukluk	-0.007	0.002	-3.73	**<0.01**
Kanektok	0.018	0.002	8.58	**<0.01**
Good News	0.024	0.001	17.11	**<0.01**
Nushagak	0.012	0.002	7.44	**<0.01**
Deshka	0.004	0.005	0.81	0.42
Kenai	0.027	0.003	10.10	**<0.01**
Copper	0.038	0.001	38.36	**<0.01**
Unuk	0.037	0.002	18.11	**<0.01**
2-ocean				
Yukon	0.009	0.003	3.65	**<0.01**
Kuskokwim	0.008	0.003	2.79	**<0.01**
Kogrukluk	0.035	0.002	15.08	**<0.01**
Kanektok	0.023	0.002	10.10	**<0.01**
Good News	0.042	0.003	15.31	**<0.01**
Nushagak	0.042	0.002	25.70	**<0.01**
Deshka	0.019	0.005	3.63	**<0.01**
Kenai	0.034	0.002	19.62	**<0.01**
Copper	0.057	0.002	29.77	**<0.01**
Unuk	0.009	0.002	3.94	**<0.01**

Bold indicates slope (log(odds)year^-1^) is significantly (*P* < 0.05) different from 0.

The analysis of the proportions of 2-, 3- and 4-ocean Chinook salmon by brood year in five datasets showed similar shifts in age-at-maturity (Fig 5) to those indicated by return year proportions (Fig 4). The declines in 4-ocean fish proportions for all 5 datasets (Kuskokwim, Nushagak, Deshka, Copper, and Unuk rivers) were significant (*P* < 0.01) (Table 4). In contrast, the regression slopes (expressed in log(odds)year^-1^) of the proportion of 3-ocean fish were positive, but regressions in only 3 of 5 of these stocks (Deshka, Copper, Unuk rivers) were significant (*P* < 0.01) (Table 4). Finally, the slopes of all of the proportions of 2-ocean fish by brood year were positive and significantly increased through time (*P* < 0.05).

**Table 4:** Beta regression of the proportions of 4-, 3-, and 2-ocean Chinook salmon by brood year returning to spawning areas from 1983 to 2012.

River system Age 4-ocean	Slope	SE	*Z*	*P*
Kuskokwim	-0.019	0.007	-2.66	**<0.01**
Nushagak	-0.036	0.010	-3.60	**<0.01**
Deshka	-0.065	0.011	-5.51	**<0.01**
Copper	-0.088	0.010	-8.53	**<0.01**
Unuk	-0.050	0.020	-2.54	**0.01**
3-ocean				
Kuskokwim	0.005	0.005	0.96	0.34
Nushagak	0.013	0.009	1.54	0.12
Deshka	0.041	0.011	3.83	**<0.01**
Copper	0.055	0.001	5.81	**<0.01**
Unuk	0.055	0.017	3.25	**<0.01**
2-ocean				
Kuskokwim	0.029	0.011	2.62	**<0.01**
Nushagak	0.050	0.012	4.295	**<0.01**
Deshka	0.017	0.009	1.978	**<0.05**
Copper	0.071	0.011	6.32	**<0.01**
Unuk	0.036	0.018	1.996	**<0.05**

Bold indicates slope (log(odds)year^-1^) is significantly (*P* < 0.03) different from 0.

The declines in length and age were consistent across populations regardless of location, commercial harvest, weir, or spawner abundance survey. No relationship between temporal trends in length-at-age or age-at-maturity was apparent with exploitation rates, which were high for the Yukon, Kanektok, and Copper rivers, medium for the Kuskokwim, Kogrukluk, and Kenai rivers, and low for the four remaining rivers (Table 1).

## Discussion

The results of our analysis of ten Chinook salmon stocks in Alaska indicate that adults have become progressively smaller in spawning areas over the past 30 years. This pattern is due to two separate trends, a downward shift in the predominant age at maturity and a decrease in age-specific size. In the early 1980s, larger, older 4-ocean fish were the predominant age class in spawning areas, but by 2012 the proportion of 4-ocean fish had progressively declined, so that 2- and 3-ocean fish were more abundant than 4-ocean fish (Figs 4 and 5). Commensurate with these shifts in age-class abundances were pervasive declines in age-specific size among the stocks. The trends that we observed in this study were not new. Previous studies also documented reductions in size over time for several individual stocks, and in some cases significant changes in size and age structure [14–16]. For example, Ricker [14] documented a significant decrease in Chinook salmon size in Alaskan troll harvests between 1960 and 1974, a trend that continued into the time frame of our analysis (1983–2012).

Before discussing the implications of these results, we offer an evaluation of the datasets that were examined. First, we could not examine the trends for males and females separately for most of the datasets, because of known problems with assigning sex to fish in marine waters before sexual traits are well developed. However, the trends we observed for the sexes combined are also likely to occur for each sex, as they did in a study of Chinook salmon in the Nushagak River where fish with spawning coloration were sampled [16]. Second, we lessened the problem by excluding early-maturing 1-ocean males (jacks) by focusing our analysis on 2-, 3- and 4-ocean fish, which constitute the bulk of returning Chinook salmon. Older age classes (≥5-ocean) were also excluded from the analysis, because they made up less than 3% on average of returning fish in all stocks, except in the Yukon River, where 7%, on average, were 5-ocean fish (1983–2011). The inclusion of jacks in our overall analysis would likely have skewed the trends that we observed, because jacks are inconsistently harvested and (or) measured and aged in commercial fisheries. Third, most datasets met parametric statistical assumptions, including a normal distribution, constant variance, and independence; hence, the trends we observed do not appear to be due to a statistical artifact. Fourth, our analysis for some areas may have been weakened to a small extent by the availability of only harvest or spawner abundance data, rather than total run (harvest + spawner abundance). In some cases, it was uncertain whether the observed changes in size and age represented actual phenotypic shifts and were not artifacts of size-biased data collection. For example, samples from gillnet fisheries may not be representative of returning fish because of size selection imposed by gill nets. Seven of the ten datasets were derived from gillnet harvests. Weir and spawner abundance survey samples may also be biased. For example, fish from the Unuk (spawning fish samples) and Deshka (weir samples) both traveled through distant size-selective commercial and sport fisheries, which may influence the sizes of fish that were sampled.

Despite these potential weaknesses, the results of our analyses convincingly show that Chinook salmon in these ten Alaska stocks have become smaller at maturity, on average, and are returning to spawning areas at a younger age. Our study extends previous observations across a broad geographic range in Alaska and includes stocks in a wide range of population and river size and with different marine migratory pathways. The datasets extended from the sub-Arctic Yukon River to spawning areas in temperate Southeast Alaska, and included populations with freshwater migrations ranging from tens to over one thousand kilometers. Size and age have been reported annually for many Chinook salmon stocks in Alaska, but these stocks as a whole have not been examined for temporal trends. Annual sample sizes for each area were large, measurement methods were consistent among datasets, and the extent of annual sampling for each stock allowed robust statistical analyses. The pattern of decline in size and age was consistent among stocks scattered over a large geographic area in Alaska and across a range of population sizes and exploitation rates.

The concordant trends among these ten Chinook salmon stocks in Alaska suggest that a common suite of large-scale mechanisms may be responsible for changes in size and age at maturity. Both genetic and environmental factors influence age at maturity. Breeding studies show large heritabilities for age at maturation that range from 49 to 57% for males and 39 to 41% for females [32]; that is, nearly half of the total variation in age at maturation is due to genetic factors. While the downward trends in age and size may have a genetic component, the drivers of these changes are more likely due to biotic and environmental factors. Several mechanisms have been suggested [14–16], including size-selective fisheries [33,34], altered growth patterns from climate [22] and marine environmental changes [35,36], nutritional restrictions in a North Pacific ecosystem increasingly saturated by large numbers of salmon [11], and density-dependent interactions with hatchery-reared salmon [37,38].

Size-selective harvests have undoubtedly led to downward shifts in age and size of some species of salmon [13,16,39] and may also be responsible, at least in part, for the patterns reported here for Chinook salmon. Chinook salmon in Alaska have been targeted in size-selective commercial, sport, and subsistence fisheries for over 100 years. Even when Chinook salmon are not targeted directly, incidental harvest in other fisheries can influence phenotypic distributions in Chinook salmon populations. For example most terminal-area fisheries predominately target sockeye salmon with smaller mesh gillnets and incidentally harvest Chinook salmon (Copper, Kenai, and Nushagak fisheries). Larger, older salmon are more abundant in terminal fisheries as they return to spawn and are susceptible to these size-selective fisheries [40].

In the present study, the patterns of overall decreased length associated with a decline in age-at-maturity and a drop in size-at-age for 4-ocean fish are consistent among areas, regardless whether the data came from commercial harvests, weirs, or escapement surveys. If size-selective fisheries were the primary mechanism responsible for the decline in fish length and age, stocks with no directed size-selective fisheries or with low exploitation rates might differ in length and age patterns from stocks subjected to high exploitation rates and directed size-selective fisheries. Instead, we found similar patterns of length and age across fisheries and exploitation rates, suggesting that size selective fisheries may not be the primary mechanism driving the declines in size and age at maturity documented here. However, we also note that our estimates of exploitation were quite coarse and leave open the possibility that even modest exploitation or size-selective harvest could, over time, result in the size and age trends observed in this study.

Environmental conditions can also influence size and age in Chinook salmon by regulating diet and growth. The relationship between growth and maturation is complex and differs among life-history stages. High nutritional condition and rapid growth at a critical early life-history stage in freshwater trigger early maturation in males as reproductively precocious jacks [41,42,43]. Nutritional condition in fry does not appear to influence the timing of maturation in adults [44].

In the marine environment, the relative influences of diet on growth and maturation are uncertain. Changes in food-web structure, associated with long-term warming trends and climatic regime shifts in the Pacific Decadal Oscillation (PDO) [19,31,45,46], may have influenced growth through shifts in the availability of forage fishes [10,23,47,48]. In addition to altered food-web dynamics, elevated temperatures may also lead to early maturation at a smaller size [12,49]. For example, temperature effects may be responsible, in part, for a latitudinal gradient in age-at-maturity of Pacific salmon in general [50]. Chinook salmon in Alaska typically return at an older age than their counterparts to the south along the North American west coast [6]. After the PDO shift in 1977, ocean conditions in Alaskan waters began to resemble the warmer conditions of southern areas, and these environmental changes may select for adults that mature as younger and smaller fish. However, the effects of temperature and diet on growth and maturation are confounded on this large geographic scale, such that it is difficult to tease apart specific influences on decadal trends.

Under the current paradigm, faster-growing salmon are expected to mature at younger ages than slower-growing fish [3,4,28,29,32,51], such that if the maturation-size threshold remains constant, size-selective fishing favors more rapidly growing and earlier maturing fish (Fig 1A in Hard et al. [9]). In contrast to our results, this model predicts an increase in the proportion of older 4-ocean fish, based on the trend of slower growth of both 3- and 4-ocean fish found here. Instead, our results show smaller fish maturing at a younger age. The mean length-at-age at maturity for 3- and 4-ocean fish declined over the time series of this analysis (Fig 2), providing evidence of either a decadal reduction in growth, or a decrease in the size threshold at which maturation occurs [52,53].

The increase in the proportion of 3-ocean fish that we observed is also counter to the prediction that rapidly growing salmon of a given cohort mature earlier [6,28,37]. If this were the case, the sizes of younger 3-ocean fish would be expected to remain unchanged or to increase, but not to decrease. We found a less-consistent pattern of declining length at age of 3-ocean fish among areas, which may be explained by the length of time these fish are in the marine environment. These fish spend 25% less time in the marine environment than do 4-ocean fish. Assuming that changes in the marine environment are driving the length at age trend, we conclude that the longer the fish is exposed to these conditions, the more the decline becomes apparent. One explanation for the patterns seen in this study is that the maturation length threshold [13] and survival to older ages have both declined so that slow-growing fish mature at a younger age.

Competition and dietary restriction, associated with density-dependent interactions in marine environments, can also influence salmon population abundance, size at age, and age at maturation [54–56]. The number of salmon in the Pacific Ocean is at an all-time high, in part, because of large-scale hatchery production across the North Pacific [57,58]. Inter- and intra-specific salmon competition can lead to slower growth rates and to reductions in the mean sizes of returning fish [11,59–61]. Beyond correlations, it has proven difficult to directly link specific biotic and environmental mechanisms to the changes observed here, because of the ocean-wide scale of these interactions and the many confounding mechanisms.

The declines in size and age of Alaska Chinook salmon reported here have implications for the long-term viability of Alaska's fisheries. Downward shifts in size at age and age at maturity affect fitness by reducing fecundity and reproductive rates [8,61–65]. Larger females generally have larger and more numerous eggs [59], both of which provide reproductive advantages [7]. Larger eggs produce larger juveniles, which tend to have higher survival rates [6]. Since size and age-at-maturity are heritable [29], selection for smaller sizes leads to a feedback loop in which younger and smaller adults produce offspring that mature earlier at smaller sizes. Change in body size may also influence spawning habitat use where larger fish occupy areas with coarser substrate that smaller fish may not be able to use. Ricker (1980) postulated that a limit to the decrease would be reached when almost all females matured as 3-ocean fish and males as 2-ocean fish. These ages at maturity represent an equilibrium between minimal sizes required to sustain spawning migrations and larger sizes needed to maximize fecundity.

It is unclear if the mechanisms responsible for selecting smaller, younger fish are likely to change in the near future so that we will again see large Chinook salmon as a significant portion of Alaskan populations. Chinook salmon returns throughout Alaska have declined in recent years, with consistent declines in run size beginning about 2007, possibly linked to a decrease in productivity from as early as brood-year 2001 [66,53]. However, compensatory population growth at low abundances and a corresponding shift in the PDO to colder conditions in Alaska [67] may reverse the trends not only in size and age at return, but also in abundance. Brood-year returns following the recent appearance of cold PDO conditions have only recently begun to return to spawn, but, since individual growth and age at maturity have strong genetic components, it might take several generations for a change to become apparent.

No specific permissions were required for this study. The State of Alaska, Department of Fish and Game has blanket authority to collect biological data to manage salmon fisheries. This report examined existing data that had been collected in the past by the Department and did not require the collection of additional samples specifically for the study. The results reported here did not involve endangered or protected species. Full details of collection and sampling methods are detailed in the manuscript. No animals were sacrificed for this study; however, some measurements were made on commercially harvested fish. No IACUC or equivalent animal ethics committee approval was needed to make the length and age measurements.

## Supporting information

Some of the AL data used in this paper are available online from the ADFG data archive at http://www.adfg.alaska.gov/CommFishR3/Website/AYKDBMSWebsite/DataTypes/ASL.aspx. Additional data were obtained from various published and unpublished sources from ADF&G.

## Supporting Information

S1 Data Chinook Length. (CSV)

S2 Data Chinook Return Year Age. (CSV)

S3 Data Chinook Brood Year Age. (CSV)
