# Correction: Changes in Size and Age of Chinook Salmon *Oncorhynchus tshawytscha* Returning to Alaska

**DOI:** 10.1371/journal.pone.0132872

**Published:** 2015-07-13

**Authors:** The PLOS ONE Staff

The Academic Editor field is missing from this paper. The Academic Editor is James P. Meador, Northwest Fisheries Science Center, NOAA Fisheries, UNITED STATES. The publisher apologizes for the error.
